# Medication administration errors and contributing factors among nurses: a cross sectional study in tertiary hospitals, Addis Ababa, Ethiopia

**DOI:** 10.1186/s12912-020-0397-0

**Published:** 2020-01-13

**Authors:** Adam Wondmieneh, Wudma Alemu, Niguse Tadele, Asmamaw Demis

**Affiliations:** 1 Department of Nursing, College of Health Sciences, Woldia University, Woldia, Ethiopia; 2 School of Nursing and Midwifery, College of Health Sciences, Addis Ababa University, Addis Ababa, Ethiopia

**Keywords:** Medication administration errors, Factors, Nurses

## Abstract

**Background:**

Unsafe medication practices are the leading causes of avoidable patient harm in healthcare systems across the world. The largest proportion of which occurs during medication administration. Nurses play a significant role in the occurrence as well as preventions of medication administration errors. However, only a few relevant studies explored the problem in Ethiopia. Therefore, this study aimed to assess the magnitude and contributing factors of medication administration error among nurses in tertiary care hospitals, Addis Ababa, Ethiopia, 2018.

**Methods:**

We conducted a hospital-based, cross-sectional study in Addis Ababa, Ethiopia. The study involved 298 randomly selected nurses. We used adopted, self-administered survey questionnaire and checklist to collect data via self-reporting and direct observation of nurses while administering medications. The tools were expert reviewed and tested on 5% of the study participants. We analyzed the data descriptively and analytically using SPSS version 24. We included those factors with significant *p*-values (*p* ≤ 0.25) in the multivariate logistic regression model. We considered those factors, in the final multivariate model, with *p* < 0.05 at 95%Cl as significant predictors of medication administration errors as defined by nurse self-report.

**Result:**

Two hundred and ninety eight (98.3%) nurses completed the survey questionnaire. Of these, 203 (68.1%) reported committing medication administration errors in the previous 12 months. Factors such as the lack of adequate training [AOR = 3.16; 95% CI (1.67,6)], unavailability of a guideline for medication administration [AOR = 2.07; 95% CI (1.06,4.06)], inadequate work experience [AOR = 6.48; 95% CI (1.32,31.78)], interruption during medication administration [AOR = 2.42, 95% CI (1.3,4.49)] and night duty shift [AOR = 5, 95% CI (1.82, 13.78)] were significant predictors of medication administration errors at *p*-value < 0.05.

**Conclusion and recommendation:**

Medication administration error prevention is complex but critical to ensure the safety of patients. Based on our study, providing a continuous training on safe administration of medications, making a medication administration guideline available for nurses to apply, creating an enabling environment for nurses to safely administer medications, and retaining more experienced nurses may be critical steps to improve the quality and safety of medication administration.

## Background

Most people around the world will take medications at some point in their life to prevent or treat illness. However, medicines do sometimes cause severe harm, disability, and even death if taken incorrectly [1]. Medication errors are the leading causes of avoidable patient harm in the health care system across the world. In African health care setting medication errors are common health problems [1, 2]. Medication administration errors (MAEs) are the most common types of medication errors posing dangerous consequences for patients, health professionals and health institutions [2, 3]. The administration of medications are primarily the nurse’s responsibility, on which spend up to 40% of their time on administering medications [4]. Nurses represent the last safety check in the chain of events in the medication administration process, and are the last safeguard of patient wellbeing [5].

Medication errors can occur at any of the phases of the medication use process: during prescribing, transcribing, dispensing and/ or administering. However, existing studies revealed that medication errors most frequently occur during the administration phase [1, 2, 6]. Most of the medications are administered by nurses [7]. The frequently perpetrated types of MAEs include wrong dose, wrong time, wrong drug, wrong route, omission of doses, wrong patient, lack of documentation, and technical errors [8–11].

To improve patient safety nurses should interrupt any medication errors before reaching the patient by adhering to the six rights of medication administration and reporting the medication administration error (MAEs) [5, 11, 12]. The six rights of medication administration are the right patient, right drug, right time, right route, right dose and right documentation [13]. Moreover, nursing and hospital managers should reduce the nursing staff workload, provide periodic training courses on the proper and safe administrations of medication and create a conducive environment for error reporting [10, 11].

The problem of MAEs is real incurring a serious threat to patient safety. Several studies and systematic reviews around the world showed the magnitude of MAE being still high [2, 6, 11, 14]. Identifying and intervening the contributing factors of MAEs is one of the best strategies proposed to improve patient safety by reducing the magnitude of MAEs. The factors are commonly identified through medication error reporting. However, studies showed that nurses are reluctant to report MAEs [8, 10, 15].

Medication errors are the most common type of medical errors that occur in hospitals, and in USA it is the eighth leading cause of death higher than car accidents, breast cancer, and AIDS combined [16, 17]. Medication errors, specially those that occur during administration are highly prevalent: each medication and each patient had at least one type of MAEs [9].

Medication errors are undoubtedly costly to patients, families, employers, hospitals, healthcare providers, and insurance companies. Patients are the primary survivor of MAEs. It profoundly affect patients in terms of morbidity, mortality, adverse drug event, additional cost and hospital stay [14]. Patients living in low-income countries experience twice as many disabilities due to medication related harm than those living in high-income countries [1]. A systematic review of adverse drug events and medication errors in African hospitals indicated that 8.4% of inpatients reported having experienced adverse drug events, while it contributed to 2.8% of admissions. Similarly, the mortality rate attributed to adverse drug events was 0.1% [2]. A direct observational study conducted in Egypt indicated 0.77% of inpatients being harmed by MAEs and needed either extended hospitalization or intervention [18]. Furthermore, in Ethiopia, 1.5% of patients experienced actual adverse drug events associated with medication errors [19].

As a second survivor, the healthcare professionals suffer from medication errors. Nurses who involved in MAEs were found to suffer from emotional distress, lack of confidence, and punitive actions, especially when the error results in substantial patient harm. They also suffer from the loss of trusts of patients and patient families who experienced MAEs [14, 16]. The health institutions as a third survivir suffer from medication errors through the increased cost of unplanned prolonged hospitalization and treatment to correct the errors. According to WHO 2017 report globally, the cost associated with medication errors has been estimated at 42 billion US Dollars annually [1, 16].

Generally, there are only a few relevant data on MAEs in developing and transitional countries, especially in Africa. In developing countries like Ethiopia with educational, economic, and trained labor problems, the issue is one of the least investigated and neglected health problems. Therefore the main aim of this study was to assess the occurrence of medication administration errors among nurses working in Addis Ababa Tertiary hospitals.

## Methods

### Study design and setting

We conducted a quantitative, institution-based, cross-sectional study in tertiary care hospitals from February to March 2018 in Addis Ababa, Ethiopia. According to food, medicine and healthcare administration and control (FMHACA), (2017) report three hospitals namely Tikur Anbesa Specialized Hospital (TASH), St. Paul’s Millennium Medical College (SPMMC) and Torhayiloch Comprehensive Specialized Hospital (TCSH) provide tertiary care. TASH has 986 Nurses with different qualification. SPMMC has 903 nurses with different qualifications. TCSH has been providing specialized service with 180 nurses.

### Population

#### Target population

All nurses working in Addis Ababa tertiary care hospitals.

#### Study sample

Those randomly selected nurses.

### Eligibility criteria

All nurses who have a minimum of diploma qualifications in nursing, a minimum of one-year work experience and involved in direct patient care were included in the study.

### Sample size determination and sampling technique

We estimated the number of nurse participants using a single population proportion formula with the consideration of the following assumptions: a MAE rate of 71% [11], the margin of error 5, 95% confidence level and non-response rate 10%. The total sample size was calculated to be 303. The study participants of each hospital were selected by using simple random sampling technique. The observational data were collected by directly observing nurses continuously for 48 h during medication administration to patients in medical, surgical and emergency department.

### Study variables

Socio-demographic characteristics of nurses and factors that contributed to MAEs were the independent variables whereas the occurance of MAE was the outcome variable.

### Sampling procedure

We started the recruitment procedure with the acquisition of the lists of all nurses working in the three selected tertiary hospitals. Then, we proportionally allotted the sample size to the hospitals number of nurses: 145 nurses to Tikur Anbesa Specialized Hospital, 132 nurses to the St. Paul’s Millennium Medical College, and 26 nurses to the Torhayiloch Comprehensive Specialized Hospital. Following the identification of the potential candidates using the inclusion criteria, we located each study units by using a simple random sampling method. Next, five trained data collectors (diploma nurses) administered the survey questionnaire to those candidates who would participate in the study. Finally, the data collectors, with the guidance of three supervisors, collected the tool back after checking its completeness and consistency.

To triangulate the data, we also conducted a continuous 48-h direct-observation of nurses while administering medications to inpatients in the medical, surgical and emergency units. Eight trained diploma nurses, with supervision by three trained BSc nurses, collected the observational data.

### Data collection instruments, and personnel

The data were collected using a structured self-administered questionnaire which was adopted from a questionnaire developed by a previous study [11]. The tool contained 43 items arranged in five sections. The first section focused on the demographic features of the participants, the second section on work-related experience, the third section on the rate and magnitude of MAEs, the forth section on the types of MAEs, and the last section on the predictors of MAEs defined by the nurse self-repot.

The direct-observation was conducted using a structured checklist adopted from the previous studies [9, 11]. It contained eight components. The observational checklist was used to gather data by observing nurses while medicating patients to assess whether they follow the six rights of medication administration or not. The questions were designed to elicit a ‘yes’ or ‘no’ response depending on the degree of nurses’ adherence to the six rights of medication administration during the process of a medication administration.

### Data quality control

We used the following measures to ensure the validity and reliability of the data: We obtained the data using two approaches (self-reporting and direct-observation: data triangulation). We adopted the survey questionnaire from the previous study [11] conducted in a very similar setting. It reported internal consistency reliability (Cronbach’s alpha coefficient) of 0.84, and a content validity index of 0.86. We randomly selected the participants, which enhances the face validity of the study. Both tools underwent an expert review. We pre-tested both tools 2 weeks ahead of the actual study. The data collectors and supervisors were recruited from hospitals that were not included in the study, by emphasizing their experience on data collection and supervision. We trained both the data collectors and supervisors on the tools and data collection procedures. We used separate data collectors and supervisors in each survey. The direct-observation was blinded: the nurses, who were observed while administering medications were not informed that they were under observation to avoid Hawthorn’s effect. Finally, the study group checked the completeness and consistencies of the tools on the spot and every night.

### Data entry, analysis, and presentation

The data were coded, cleaned, edited and entered into Epi data version 4.2 and exported to SPSS window version 24 for analysis. All variables with *P* ≤ 0.25 in the bivariable analysis model were included in the final model of multivariable analysis in order to control all possible confounders. Model fitness was checked with the Hosmer-Lemeshow test. Adjusted odds ratio with 95% CI was estimated to identify the factors associated with MAEs using multivariable logistic regression analysis. Level of statistical significance was declared at *p*-value < 0.05.

## Result

### Demographic characteristics

Two hundred and ninety-eight (98.3%) nurses completed and returned the survey questionnaire. Of these, 198 (66.4%) were female, 217 (72.8%) were single, and 260 (87.2%) had a BSc Degree in Nursing Science (Table 1).

**Table 1:** Demographic characteristics of nurses in tertiary hospitals, Addis Ababa, Ethiopia, 2018

No.	Variables	Response	Frequency (*n* = 298)	Percentage (%)
1	Age	20–24 years	98	32.9
		25–29 years	146	49
		30–34 years	28	9.4
		≥35 years	26	8.7
		Mean = 27.2 years, SD =5.1		
2	Sex	Female	198	66.4
		Male	100	33.6
3	Marital status	Single	217	72.8
		Married	79	26.5
		Others^a^	2	0.7
4	Educational status	Diploma nurse	25	8.4
		BSc nurse	260	87.2
		MSc nurse	13	4.4
5	Educational award from	Government institution	168	56.4
		Private institution	130	43.6

Others^a^ = divorced and separated

### Work-related nurse characteristics

The median work experience of the respondent was 2 years (ranged from one to 33). From the 298 nurses, 114 (38.3%) worked in the day-shift, 166 (55.7%) reported using guidelines to administer medications, 185 (62.1%) were not trained on safe administration of medications, and 161 (54%) faced interruption while administering medications (Table 2).

**Table 2:** Work-related characteristics of nurses in tertiary hospitals, Addis Ababa, Ethiopia, 2018

No.	Variables	Response	Frequency (*n* = 298)	Percentage (%)
1	Work experience	< 10 years	269	90.3
		≥ 10 years	29	9.7
		Median = 2 years		
2	Duration in the present unit	≤ 3 month	28	9.4
		4–5 month	26	8.7
		≥ 6 month	244	81.9
3	Duty shift	Day shift	114	38.3
		Night shift	79	26.5
		Alternative shift	105	35.2
4	Nurse to patient ratio	1–6	72	24.2
		7–10	106	35.6
		> 10	120	40.3
5	Take training in MA practice	Yes	113	37.9
		No	185	62.1
6	Have guideline for MA	Yes	166	55.7
		No	132	44.3
7	Faced interruption during MA	Yes	161	54
		No	137	46
8	Communicate with another nurse before MA	Yes	250	83.9
		No	48	16.1

### Prevalence and types of medication administration error

Two hundred and three (68.1%) nurses were involved in MAEs in the previous 12 months. Of these, 77 (37.9%) claimed making MAE only once while 119 (58.6%) made MAEs 2–3 times and the remaining 7 (3.4%) made MAEs more than four times.

Failure to administer medications at the right time was the most 117 (57.8%) frequently breach in the rights of medication administration followed by breach in documentation 50 (24.8%) and dose 46 (22.5%) respectively. The administration of a medication through a route other than the ordered route was the fourth common incident: 38 (18.5%) nurses admitted administering medications through wrong routes. Twenty four (11.7%) nurses were administered the wrong medications; and 18 (8.7%) nurses were administered medications to the wrong patients.

### Observational checklist result

To triangulate the result of self-administered questionnaire, observational data were collected by observing nurses during medication administration continuously for 48 h. The single medication administered by a nurse was considered as a single dose and totally 225 doses of medications were observed. Most (96%) of the 225 directly observed doses involved a breach in the recipients’ safety at least once (Table 3).

**Table 3:** The level of adherence to the six rights of medication administration, a direct observational survey in tertiary hospitals, Addis Ababa, Ethiopia, 2018 (*n* = 225 doses)

No.	Variable	Response	Frequency (*n* = 225)	Percentage (100%)
1	Wash hands/ hand rub before the procedure	Yes	54	24.0
		No	171	76.0
2	Wear/ change glove	Yes	131	58.2
		No	94	41.8
3	Give medication to the right patient	Yes	191	84.9
		No	34	15.1
4	Administer the right medication	Yes	188	83.6
		No	37	16.4
5	Administer the right dose	Yes	173	76.9
		No	52	23.1
6	Administer through right route	Yes	193	85.8
		No	32	14.2
7	Administer medication at the right time	Yes	147	65.3
		No	78	34.7
8	Document necessary information	Yes	108	48
		No	117	52
9	Distractors	Yes	60	26.7
		No	165	73.3

### Predictors of medication administration error

Binary logistic regression was done to identify factors associated with medication administration errors. In multivariate logistic regression analysis factors that were significantly associated with MAEs were work experiences, availability of guideline for MA, take training, interruption during MA and Night shift.

Nurses who didn’t used guidelines for medication administration were two times more likely made MAEs than those who had used guidelines for medication administration [AOR = 2.07, 95% (CI: 1.06–4.06)]. Likewise, nurses who had not been trained on safe administration of medications were three times more likely made MAEs than those who had been trained [AOR = 3.16; 95% CI (1.67, 6.00)]. Nurses who were interrupted while administering medications were 2.42 times more likely committed MAEs than those who weren’t interrupted administering medication [AOR = 2.42, 95% CI (1.30, 4.49)]. Regarding work experience, nurses who had less than 10 years work experience were almost 6.5 times more likely committed MAEs than those nurses who have 10 years and above work experiences [AOR = 6.48; 95% CI (1.32, 31.78)]. Finally, those nurses who were in night shift duty were five times more likely made MAEs than those nurses working in daytime shift [AOR = 5, 95% CI (1.82, 13.78)] (Table 4).

**Table 4:** Bivariate and multivariable logistic regression analysis of factors associated with MAE in tertiary hospitals, Addis Ababa, Ethiopia, 2018

Variables	Medication administration error		Bivariate logistic regression	Multivariable logistic Regression	*P*-Value
	Yes	No	COR [95%CI]	AOR [95%CI]	
Age					
20–24 years	77 (37.9%)	21 (22.1%)	5 [2.0–12.49]	0.57 [0.10–3.30]	0.53
25–29 years	99 (48.8%)	47 (49.5%)	2.87 [1.22–6.73]	0.35 [0.06–1.95]	0.23
30–34 years	16 (7.9%)	12 (12.6%)	1.8 [0.62–5.35]	0.41 [0.08–2.09]	0.28
≥ 35 years	11 (5.4%)	15 (15.8%)	1.000	1.000	
Educational award from					
Gov’t institution	106 (52.2%)	62 (65.3%)	1.000	1.000	
Private institution	97 (47.8%)	33 (34.7%)	1.70 [1.04–2.85]	1.37 [0.73–2.6]	0.32
Work experience					
< 10 years	193 (95.1%)	76 (80%)	4.80 [2.15–10.85]	6.48 [1.32–31.78]	0.021
≥ 10 years	10 (4.9%)	19 (20%)	1.000	1.000	
Duration in the specific unit					
≤ 3 month	22 (10.8%)	6 (6.3%)	1.70 [0.66–4.34]	1.57 [0.55–4.54]	0.39
4–5 month	14 (6.9%)	12 (12.6%)	0.54 [0.24–1.22]	0.54 [0.20–1.48]	0.23
≥ 6 month	167 (82.3%)	77 (81.1%)	1.000	1.000	
Duty shift					
Day shift	56 (27.6%)	48 (50.5%)	1.000	1.000	
Night shift	73 (36%)	6 (6.3%)	10.4 [4.17–26.1]	5 [1.82–13.78]	0.002
Alternate shift	74 (36.4%)	41 (43.2%)	1.54 [0.9–2.66]	1.47 [0.78–2.76]	0.22
Took training					
Yes	56 (27.6%)	57 (60%)	1.000	1.000	
No	147 (72.4%)	38 (40%)	3.9 [2.35–6.57]	3.16 [1.67–60]	< 0.001
Have guideline					
Yes	96 (47.3%)	70 (73.7%)	1.000	1.000	
No	107 (52.7%)	25 (26.3%)	3.1 [1.83–5.32]	2.07 [1.06–4.06]	0.03
Faced interruption					
Yes	124 (61.1%)	37 (39%)	2.46 [1.49–4.05]	2.42 [1.30–4.49]	0.005
No	79 (38.9%)	58 (61%)	1.000	1.000	
Communicate					
Yes	165 (81.3%)	85 (89.5%)	1.000	1.000	
No	38 (18.7%)	10 (10.5%)	1.96 [0.93–4.12]	1.85 [0.72–4.77]	0.19
Report error					
Yes	38 (18.7%)	11 (11.6%)	1.000	1.000	
No	165 (81.3%)	84 (88.4%)	0.57 [0.27–1.67]	0.68 [0.29–1.57]	0.37

Key:

*COR* Crude Odd Ratio

*AOR* Adjusted Odd Ratio

## Discussion

One of the strategies to safeguard patient’s safety and improve the quality of nursing care is the proper administration of medications. However, the finding of this study showed that the magnitude of MAE was high (68.1%) in Addis Ababa tertiary hospitals. More than half of the medication errors occurred during administrations of medication and nurses are in the front line for the administrations of medication [20].

The prevalence of MAE in this self-reported study is relatively consistent with studies conducted in Iran teaching hospital (68.5%), Indian tertiary hospital (68.5%), three university hospitals of South Korea (69.6%), and a study review conducted in Iran (70%) [20–23]. On the other hand, the result of this finding was higher than those studies conducted in Turkey state hospital (61.7%) and a systematic review conducted in England (54.4%) [3, 24]. This difference might be due to a difference in the number of hospitals and number of researched clinical departments, in which some of the above studies were conducted in a single hospital and some studies were conducted in a single department. Furthermore, the above studies were conducted in developed countries, in which better computerized prescribing and recording system, high quality of health care services, voluntary error reporting and follow up are conducted.

The result of this study was higher than a study conducted in Felege Hiwot referral hospital, which was 56.4% [9]. The plausible justification for the difference might be the variation in the number of hospitals and researched clinical units. The above study was conducted in a single hospital inpatient department only. Additionally, the previous study used convenient sampling technique. However, the finding of this study is slightly lower than a study done in two public hospitals of southern Ethiopia (71%) [11]. The possible explanation for this difference might be due to variation in the number and type of hospitals. The previous study was conducted in the two southern Ethiopia public hospitals. Additionally, there is a difference in sample size.

In this study, a total of 225 doses of medication administrations were observed. From these, 96% involved at least one MAE. This finding is relatively consistent with studies conducted in Felege Hiwot referral hospital (98.1%) and two public hospitals in southern Ethiopia (99.7%) [9, 11]. On the other hand, the result of this study is three folds higher than a study conducted in the emergency department of Accra tertiary hospital (27.2%) and more than two folds higher than a study conducted in the medical ward of Ain Shames university hospital in Egypt (37.8%) [17, 25]. The difference might be due to variation in the study settings, in which the above studies conducted in a single hospital and in a single department. However, this study was conducted in all inpatient units of three hospitals.

This study revealed that magnitude of MAEs was considerably high in Addis Ababa tertiary hospitals. This indicates that the quality of nursing care in relation to medication administration did not appear to be up to the standard. The safety of the patient during medication administration was poorly maintained. Medication administration errors of this magnitude are likely to result in harming the patient and may erode public confidence in nursing care. Several studies and systematic reviews around the world in different countries reported likewise high magnitude of MAEs [2, 3, 6, 18].

The findings of both types of study showed that MAEs were a common health problem in the hospitals under study. The magnitude of MAE in self-reported study and observational study was 68.1 and 96% respectively. This result indicated that some participants made MAE but not report in the self-reported questionnaire. Similarly in this self-reported study 83.4% of nurses were not report medication errors to the concerned body. Furthermore different literatures also showed that majority of the medication errors were not reported [11, 24, 26].

Wrong time error (58.7%) was the most frequent type of MAE detected in this study. This finding indicates that more than half of the medications were not administered at ordered time. When medications are not administered at the regularly scheduled time the patient may develop toxicities or resistance to the drugs. The finding of this study was similar to a study done in two southern Ethiopia hospitals (58.5%) [11]. However, it was much lower than a study conducted in France (72.6%) [27]. The difference likely to be due to the difference in the study setting (the previous study was conducted in a specified ward, while this study was conducted in all clinical departments of three hospitals). Additionally, there was a difference in data collection method. (The study in France used a direct observational method only. Whereas; this study used both a direct observational and self-reporting method).

On our study interruption during medication administration, lack of work experience and unavailability of a guideline for medication administration were significantly associated with MAEs. These findings were supported by studies conducted in Ethiopia Felege Hiwot Referral hospital and two public hospitals in southern Ethiopia [9, 11]. A similar study conducted in Turkey indicated that interruption by telephone and questions being asked during medication administration were found to have contributed to MAEs [24]. Medication preparation and administration need concentration. Interruption occurs when a nurse is preparing and administering a medication, which could lead to an error. Interruption of these activities may lead to cognitive failure in relation to working memory and attentiveness. Moreover making the environment conducive for nurses prior to preparation and administrations of medication may reduce medication errors. The finding of this study showed that work experiences of nurses were significantly associated with MAEs. Studies conducted in Felege Hiwot Referral hospital and emergency department of southern Iran hospital indicated that there is a significant association between work experience and MAEs [9, 21]. Medication administration is one of the nursing practices and improved through experience. When nurses with experience can improve their skill and gain greater knowledge on safe medication administration practice. Moreover experienced nurses are familiar with different medications and procedures.

Lack of training and unavailability of guideline for safe medication administration practice were also significantly associated with medication administration errors. This finding is supported by WHO 2017 medication without harm strategies. In 2017 WHO develops strategies to improve patient safety through reductions of medication error up to 50% in the next 5 years from 2017 to 2022 [1]. From these strategies provide a guideline and strengthen health professionals capacity through skill building are the major ones. On the other hand, a study done in Egypt showed that MAEs was highly prevalent and all nurses had not taken training courses on safe medication administration practices and they haven’t policies and procedures for medication administration [17]. Providing training is mandatory for medication error reduction because there is the discovery of the new disease, new medication, and new administration techniques. So to combine nursing practice with evidence training may serve as a bridge. Additionally, availability of guideline for medication administration may improve the quality of nursing care and reduce MAEs.

This study showed that medication administration time was significantly associated with MAEs. The odds of MAE among nurses administering medication during the day and night shift was 5:1[AOR = 5, 95% CI (1.82, 13.78)]. This finding is supported by studies conducted in Felege Hiwot referral hospital of Ethiopia and Ain Shams University hospital of Egypt [9, 17]. Nurses during night time may experience sleep deprivation, loss of concentration and exhaustion. Nurses use different strategies like Exercise for 30 min, moderate caffeine consumption before night and taking nap to decrease sleep disturbance and increase attention during the night shift.

Reporting medication administration errors are just a tip of the iceberg to reduce medication administration errors. However, the findings of this study indicated that 16.4% of participants reported MAEs. The result of this study is lower than studies conducted in Iran (55%), two public hospitals in southern Ethiopia (24.7%) and University of Gonder referral hospital (29.1%) [10, 11, 26]. The difference might be due to fear of blame for the reported errors in the working unit, variation in the administration support and encouragement, a variation of nurses willingness to report errors and variations in the nurse to patient ratio. Additionally, there is a difference in the study setting.

### Limitations of the study

- ➢ The finding of this study is inferred to only tertiary hospitals found in Addis Ababa due to constraints of time and fund.
- ➢ As it was a cross-sectional study, we couldn’t draw a cause and effect relationship between the MAEs and the identified factors.
- ➢ Finally the study was based on self-reported information and direct observation that may be prone to reporting bias and observer bias

## Conclusion

In conclusion, based on our findings, the safety of inpatients receiving drug therapy in the studied hospitals was frequently breached. The provision and adherence to guidelines during medication administration, creating a conducive environment and training nurses on the safe administration of medication are critical to ensure the safety of patients during medication administration.

## Data Availability

All related data has been presented within the manuscript. The datasets used and/or analyzed during the current study are available from the corresponding author upon a reasonable request.

## Abbreviations

AIDS: Acquired Immuno-Deficiency Syndrome
AOR: Adjusted Odds Ratio
CI: Confidence Interval
COR: Crude Odd Ratio
ICU: Intensive Care Unit
IV: Intravenous
MAE: Medication Administration Error
ME: Medication Error
SAQ: Self-Administered Questionnaire
SD: Standard Deviation
SPMMC: St. Paul’s Millennium Medical College
SPSS: Statistical Program for Social Sciences
TASH: Tikur Anbesa Specialized Hospital
TCSH: Torhayiloch Comprehensive Specialized Hospital
US: United States
WHO: World Health Organization
